# Characterisation of iron oxide encrusted microbial fossils

**DOI:** 10.1038/s41598-020-66830-z

**Published:** 2020-06-18

**Authors:** Alan Levett, Emma J. Gagen, Llew Rintoul, Paul Guagliardo, Hui Diao, Paulo M. Vasconcelos, Gordon Southam

**Affiliations:** 1 School of Earth and Environmental Sciences, University of Queensland, Brisbane, Queensland 4072 Australia; 2 Central Analytical Research Facility, Institute of Future Environments, Queensland University of Technology, Brisbane, Queensland 4001 Australia; 3 Centre for Microscopy, Characterisation and Analysis, University of Western Australia, Perth, 6009 Western Australia Australia; 4 Centre for Microscopy and Microanalysis, University of Queensland, Brisbane, 4072 Queensland Australia

**Keywords:** Biogeochemistry, Environmental sciences, Planetary science, Nanoscience and technology

## Abstract

Robust methods for the characterisation of microbial biosignatures in geological matrices is critical for developing mineralogical biosignatures. Studying microbial fossils is fundamental for our understanding of the role microorganisms have played in elemental cycling in modern and ancient environments on Earth and potentially Mars. Here, we aim to understand what promotes the fossilisation of microorganisms after the initial stages of biomineralisation, committing bacteriomorphic structures to the geological record within iron-rich environments. Mineral encrusted cell envelope structures were routinely identified within a goethite-rich vein that cross-cut the saprolite (iron ore) of a weathered banded iron formation (BIF) system in the Quadrilátero Ferrífero, Brazil. The preservation of potential organic and mineralogical biosignatures associated with these fossils was characterised using the following high-resolution analytical techniques: scanning and transmission electron microscopy, focused ion beam scanning electron microscopy, nanoscale secondary ion mass spectrometry, synchrotron-based Fourier transform infrared spectroscopy and Raman spectroscopy. Electron microscopy demonstrated that mineral nucleation associated with a range of cell envelope structures typically followed the extant cell templates. These biologically-influenced iron-rich minerals are microcrystalline with minimal secondary growth. In contrast, intracellular mineralisation formed larger minerals that grew inward from the cell membrane to infill intracellular voids after cell death. A three dimensional reconstruction of encrusted cell envelopes in a fossilised biofilm suggests that microorganisms may be able to replicate, during the initial stages of mineralisation. Carbon and nitrogen signatures are preserved associated with the cell envelope structures; however, there were no conclusive mineralogical biosignatures associated with the mineralised cell envelopes highlighting the classical importance of morphology and elemental biosignatures in determining the biogenicity of bacteriomorphic structures.

## Introduction

In 1996, the geoscience community was challenged with a question: What evidence is required to prove the existence of life^1^? Characterisation of the ALH84001 meteorite presented 20 nm diameter tubular nanofossil structures, indirect organic signatures and mineral formation anomalies as evidence for past life. Intense scrutiny of these controversial results has led to questions regarding the robustness of these signatures as evidence of life; however, the work has been instrumental in stimulating scientists to better understand microbial fossilisation and the array of microbial biosignatures that may be preserved^2^.

The surface of Mars is extremely inhospitable for life as we know it, with records of life on Mars likely to be restricted to mineralogical biosignatures^3^. Therefore, in-depth characterisation of microbial fossils here on Earth is required to develop new biosignatures that may be preserved in the geological record, particularly mineralogical biosignatures. Low temperatures and pressures, little-to-no atmospheric protection from ionising radiation and oxidising geological conditions provide little optimism for finding extant life. Future Mars Rover missions are being designed to collect, encapsulate and store samples from below the surface (up to 2 m depth) for later collection and transportation back to Earth. The potential success of such rover-based missions requires identifying key near-surface environments on Earth conducive to microbial fossilisation^4^. This article responds to a call for additional work and understanding of microbial fossilisation in pore and fracture filling near-surface environments^4^.

Here, we have correlated a range of high-resolution analytical techniques to characterise well-preserved iron oxide encrusted microbial cell envelopes fossilised in vein structures below the surface (~15 m depth) to aid in the search for robust microbial biosignature targets. Studying the mineralogy associated with microfossils assists in constraining the environments in which microorganisms existed and their role in altering the biogeochemistry of their local environment. The continued development of nano- and microscale analytical techniques provides scientists with an increasingly large toolkit to more effectively characterise bacteriomorphic structures and determine their biogenicity. This article offers insights into the effectiveness of various analytical methods when assessing biogenicity of bacteriomorphic structures and the development of robust biosignature targets. In addition, this manuscript serves a timely reminder that we, as a scientific community, must maintain high standards for what we accept as microbial fossils as set out by Westall^5^ to avoid ambiguity in the literature.

## Materials and Methods

Naturally fossilised microorganisms were identified within a goethite-rich vein that cross-cut an iron ore deposit in the Quadrilátero Ferrífero, Minas Gerais, Brazil from a depth of approximately 15 m^6,7^. The goethite-rich vein formed during the weathering of the hosting BIF and the vein minerals, likely to be carbonate or sulphide^7^. (U-Th)/He geochronology of goethite fragments from this sample (G-12-11) yield precipitation ages ranging from 40 to 30 Ma^8^, demonstrating the preservation of these biosignatures over geologic time. Monteiro^9^ (Chapter Six) discusses the complete paleoclimate conditions in the Quadrilátero Ferrífero and highlights temperature effects on the oxygen isotope concentrations of goethite minerals are minor, with mineral alteration occurring at low temperatures.

### Scanning electron microscopy

Polished petrographic thin sections (100 µm thick) were prepared by dehydrating the vein rock fragment at 40 °C overnight and embedding in EpoxiCure 2 epoxy. Thin sections, coated in 10 nm iridium using a BAL-TEC MSC-010 sputter coater, were examined using a JEOL7100 scanning electron microscope in backscattered electron mode at an accelerating voltage of 15 kV to identify fossilised microorganisms and regions of interest for further analysis.

### Focused ion beam scanning electron microscopy

#### Sample preparation

For all analyses except Raman spectroscopy, sample preparation of mineralised cell envelopes was performed using a FEI Scious focused ion beam scanning electron microscope (FIB-SEM) DualBeam system with lift-out capabilities to extract lamella. For nanoscale secondary ion mass spectrometry (NanoSIMS) and synchrotron-based Fourier transform infrared spectroscopy (FT-IR), procedures outlined in were followed for sample preparation. Briefly, a large lamella, 40 µm × 80 µm and 4 µm thick, was prepared by upscaling the typical TEM lamella preparation technique^10^. The heterogeneous nature of the fossilised biofilms and the fact that lamellae were extracted vertically from the thin section meant the preparation of a quality section was somewhat serendipitous. For transmission electron microscopy, a standard-sized lamella (approximately 4 × 4 µm and 0.1 µm thick) was prepared from a region previously used to remove a large lamella, thereby offering a view into the ‘subsurface’ in these thin sections.

#### Three dimensional (3D) visualisation of fossilised biofilms

Microfossils encrusted in iron oxides were imaged using slice-and-view technology on the FIB-SEM for high-resolution 3D imaging. A platinum deposition was used to protect an area of approximately 25 × 25 µm containing abundant fossilised microorganisms. A gallium probe of 30 nA was used to mill trenches surrounding the area of interest to prevent shadowing effects during imaging. Slices with a thickness of 100 nm and approximately 20 µm in depth were milled using a 3 nA gallium ion probe. A series of backscattered scanning electron (BSE) micrographs were accumulated using an accelerating voltage of 2 kV during the automated slice-and-view process. Amira 6.1. (FEI Visualisation Sciences Group) software was used to assemble the electron micrographs to reconstruct the three-dimensional structure of the fossilised microorganisms.

### Nanoscale secondary ion mass spectrometry (NanoSIMS)

High-resolution elemental maps were acquired using the CAMECA NanoSIMS 50 at the University of Western Australia. Elemental maps were acquired using a Cs^+^ ion beam, which was focused to a diameter of 50–60 nm, using a current of 0.3 pA. The primary Cs^+^ ion beam was used to sputter the following secondary ions: ^12^C_2_^−^, ^12^C^14^N^−^, ^27^Al^16^O^−^ and ^56^Fe^16^O^−^. High-resolution elemental micrographs were acquired from a 12 × 12 µm region of interest by rastering the beam with a dwell time of 80 msec per pixel at a resolution of 256 × 256 pixels. To remove any surface contamination and implant Cs^+^ into the sample’s surface, lamella were pre-sputtered for 10 min using the Cs^+^ ion beam. Semi-quantitative maps are presented as grayscale intensity maps, with white regions indicating a higher relative abundance. FIJI software using the OpenMIMS plugin was used for data analysis.

### Infrared microspectroscopy

#### Fourier transform infrared (FT-IR) microspectroscopy

To determine the nature of organic biosignatures preserved associated with the encrusted cell envelope structures, a FIB lamella was analysed using infrared microspectroscopy at the Australian Synchrotron (Clayton, Australia). A Bruker V80v FT-IR spectrometer with a photovoltaic liquid nitrogen cooled mercury-cadmium-telluride (MCT) detector system (Bruker Optik GmbH, Ettlingen, Germany) was operated in transmission mode with a beamsize of 4.17 µm to acquire mid-infrared spectra (3800–900 cm^−1^). Spectra were obtained by accumulating 128 scans with a resolution of 4 cm^−1^. The sample was moved in a raster fashion with a step size of 2 µm. Data were processed using OPUS 8.0 software (Bruker Optik, GmbH, Ettlingen, Germany). To produce the infrared map, all spectra were integrated between 2910 and 2940 cm^−1^ to highlight regions enriched with aliphatic hydrocarbon moieties of CH_2_. The infrared map is displayed as a contoured heat-map, with the largest integrated absorbance areas represented by pink regions.

#### Raman microspectroscopy

Raman spectra from a petrographic thin section were acquired using a WITec Alpha 300 series Raman equipped with a 532 nm laser, operated at 0.53 mW. The laser was focussed with a Zeiss Plan-Neofluar 100×/1.30 Oil objective using Zeiss Immersol 518 F immersion oil. The region of interest was mapped by raster motion in X and Y increments of 0.25 μm with a dwell time of 12 s. To generate mineral maps, Classical Least Squares (CLS) methods used each spectrum as a linear combination of individual constituent spectra, plus error. Using this method, CLS can decompose a spectrum into a set of constituent scores of individual components to produce semi-quantitative mineral maps. Reference spectra of goethite and lepidocrocite for CLS calculations were extracted from within the data set (Supplementary Table 1)^11^. The internal standard spectra and representative spectra of goethite and lepidocrocite are provided in Supplementary Figs. 2–3, respectively. To limit the interference by broad fluorescence, the background was removed from all spectra prior to analysis. The baseline was removed using the rolling circle filter with a nominal diameter of 250 as implemented in the “shape” function of the Witec Project 4.1 software^12^. Data analysis, including CLS calculations, and instrument control were performed using Project Four software and WITec Control Four, respectively.

### Transmission electron microscopy

Rock samples, prepared for transmission electron microscopy using the focused ion beam scanning electron microscopy, were examined using a FEI Technai F20 field emission scanning transmission electron microscope (FEG-S/TEM) operated in bright field mode at 200 kV.

## Results

**Figure 1:**
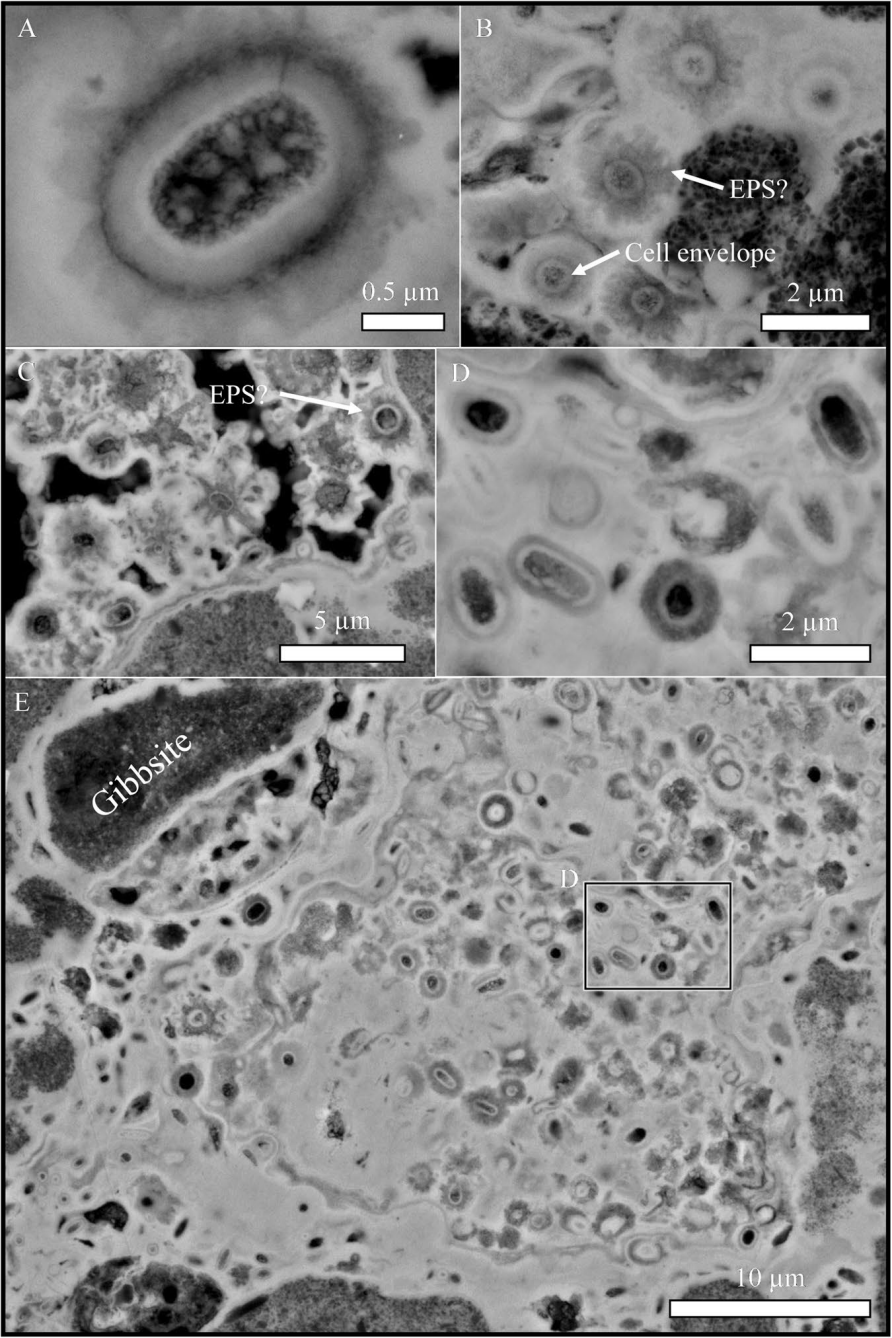
Backscattered scanning electron micrographs highlight the preservation of the cell envelopes structures (**A**), possibly including extracellular polymeric substances (**B**). Microfossils are typically cocci-shaped (**C**) but occasionally rod-shaped microfossils are preserved (**D**). Fossilised microorganisms can infill relatively large pore spaces in the subsurface (**E**).

**Figure 2:**
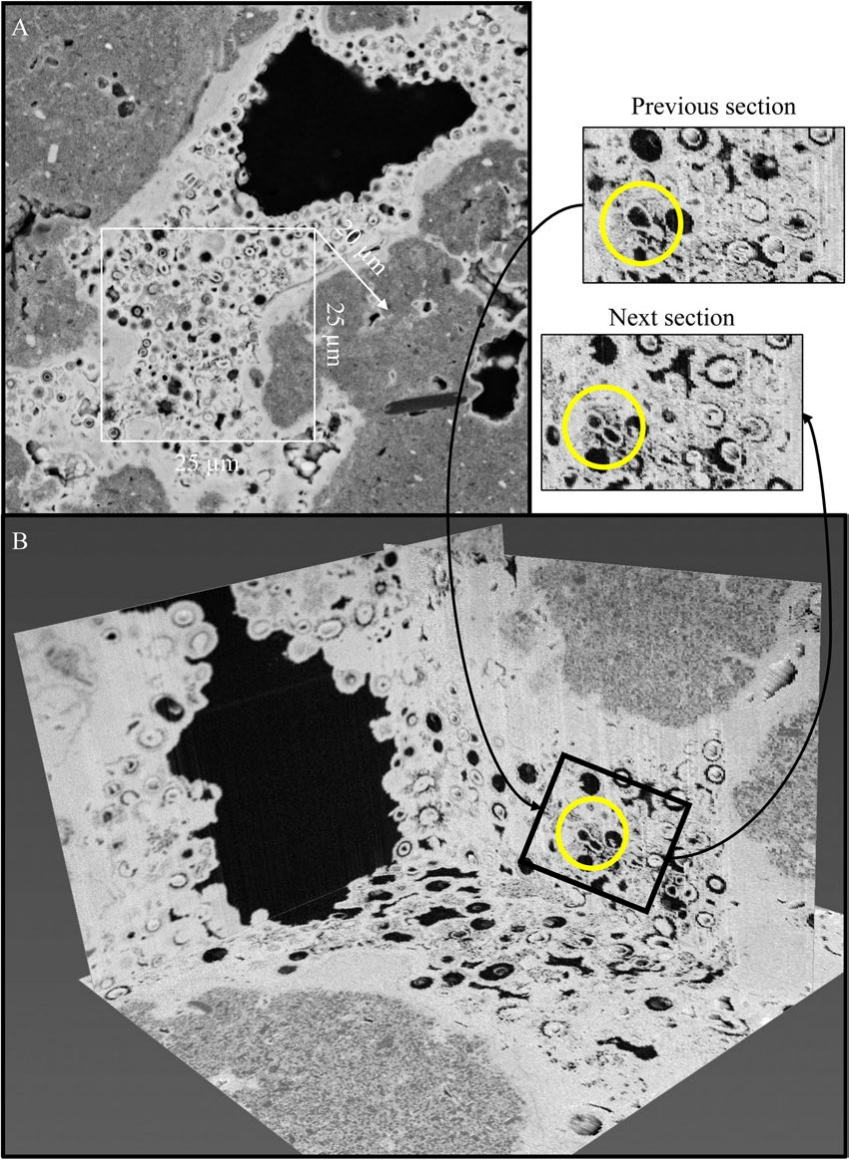
(**A**) Backscattered scanning electron micrograph of fossilised biofilm from which 3D reconstruction was created (see Video 1). (**B**) Orthogonal slice-view of the fossilised biofilm highlighting textures that may indicate cells can replicate even when partially mineralised.

Scanning electron microscopy revealed the extraordinary preservation of cell capsule structures and potentially extracellular polymeric substances (EPS; Fig. 1). A 3D reconstruction of the fossilised biofilm (Fig. 2) highlights that as minerals continue to precipitate in association with the microorganisms, preserving the encrusted cell envelope structures. This process occurs in generations; secondary iron oxide minerals infill the extracellular regions between preserved cell envelopes as new microorganisms are fossilised within the pore space. As the microorganisms are continuously fossilised, the pore space of the fracture is infilled (Video 1).

**Figure 3:**
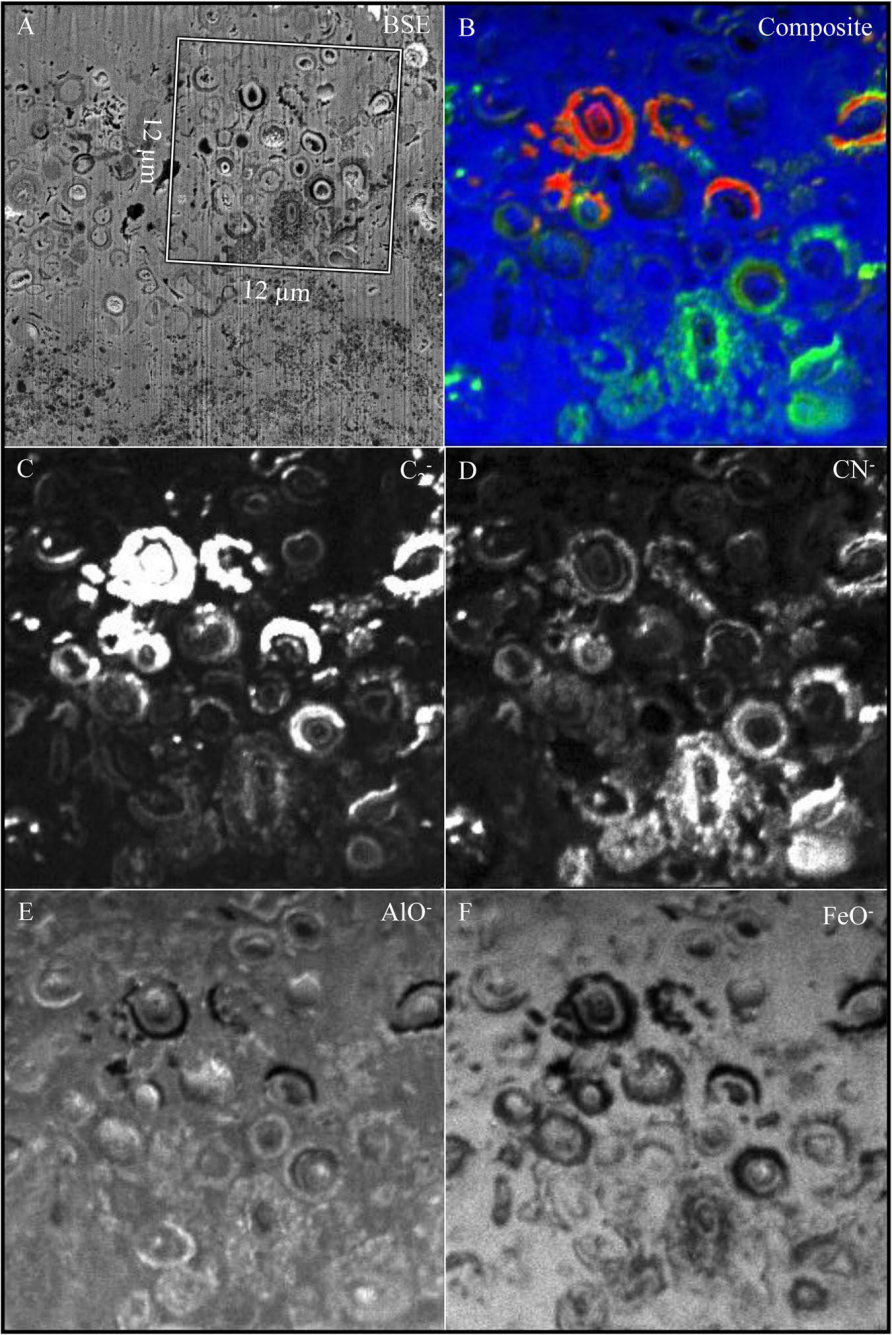
(**A**) Backscattered scanning electron micrograph of FIB lamella highlighting the region mapped using NanoSIMS (white square). (**B**) Composite image highlighting the relative distributions of carbon (red), nitrogen (green) and iron (blue). Carbon (**C**), nitrogen **(D**), aluminium oxide (**E**) and iron oxide (**F**) NanoSIMS elemental micrographs highlighting the preservation of carbon and nitrogen associated with the cell envelope structures.

**Figure 4:**
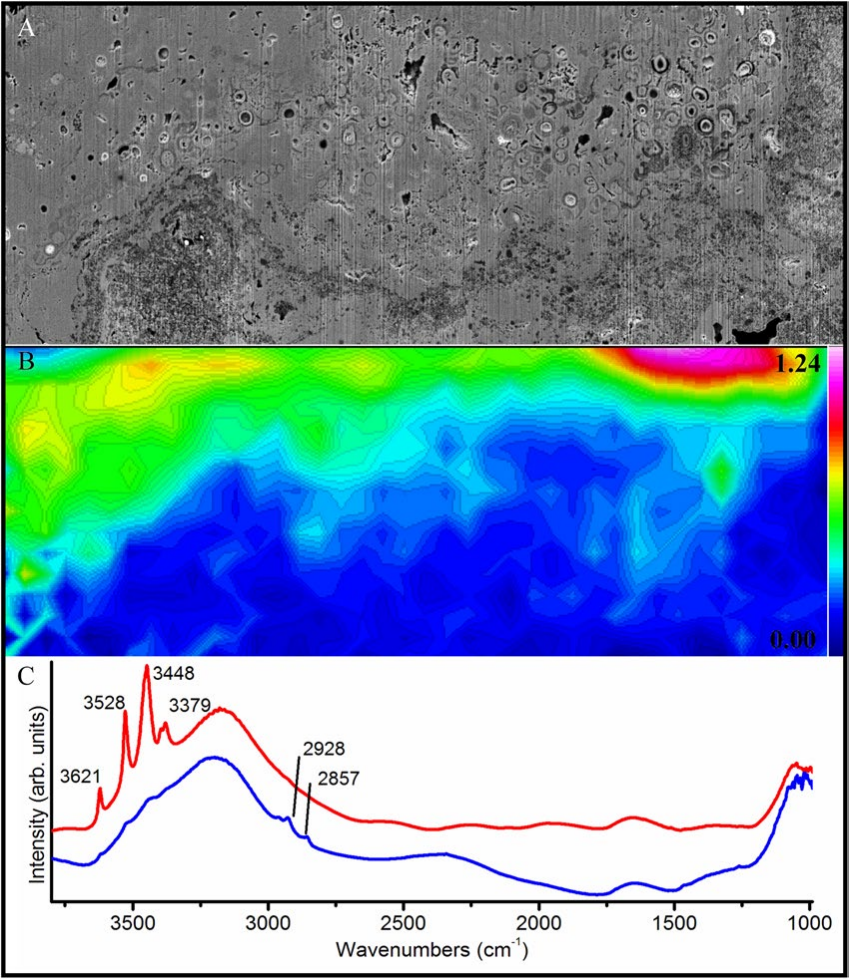
(**A**) Backscattered scanning electron micrograph of a FIB lamella highlighting the region mapped using synchrotron-based Fourier transform infrared spectroscopy (FT-IR). (**B**) Contoured heatmap generated by integrating all spectra between 2910 and 2940 cm^-1^, corresponding to the antisymmetric stretch of aliphatic moieties (CH_2_). (**C**) Infrared spectrum of a region containing methylene moieties (blue spectrum), associated with the microfossil-rich regions of the lamella. Red spectrum highlights the minerals around microfossils are gibbsite-rich.

NanoSIMS analysis revealed preserved organic carbon and nitrogen associated with the cell envelope structures of microfossils (Fig. 3). Synchrotron-based FT-IR analysis highlighted the enrichment of organic biosignatures associated with regions containing microfossils, but it was not able to resolve individual cell envelope structures (Fig. 4). The preserved organic biomarkers appeared to be aliphatic methylene moieties (CH_2_), highlighted by the bands at 2857 and 2928 cm^−1^ (Fig. 4), corresponding to the antisymmetric and symmetric stretching of saturated hydrocarbons, respectively. Microfossils were preserved within an iron-rich matrix surrounding gibbsite grains, highlighted by bands at 3379, 3448, 3528, 3621 cm^-1^ (Fig. 4).

**Figure 5:**
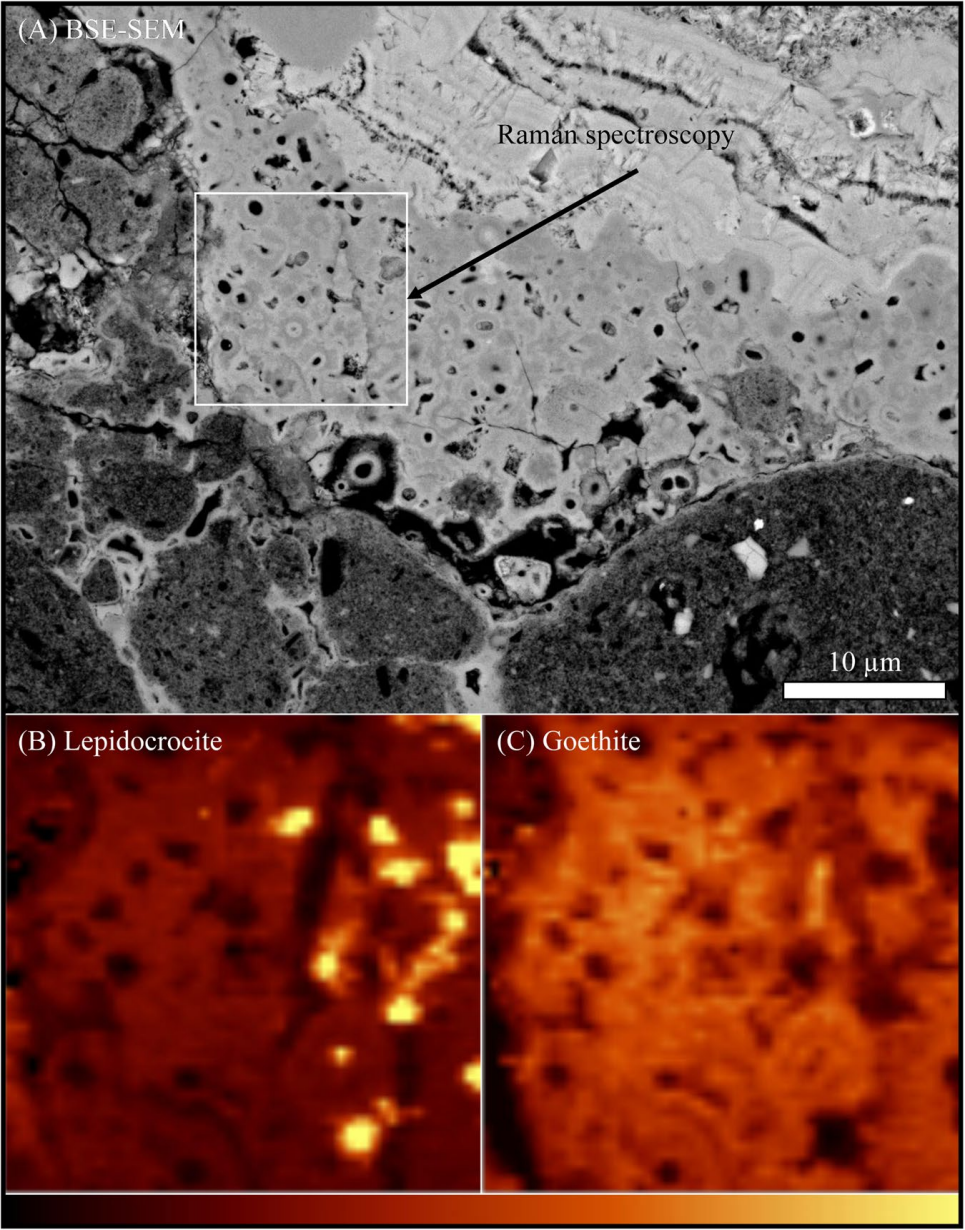
**(A**) Backscattered scanning electron micrograph highlighting region mapped using Raman spectroscopy, which revealed iron oxide minerals associated with microfossils are minor lepidocrocite (**B**) and goethite (**C**). Lepidocrocite is enriched in the extracellular regions between fossilised cell envelopes.

**Figure 6:**
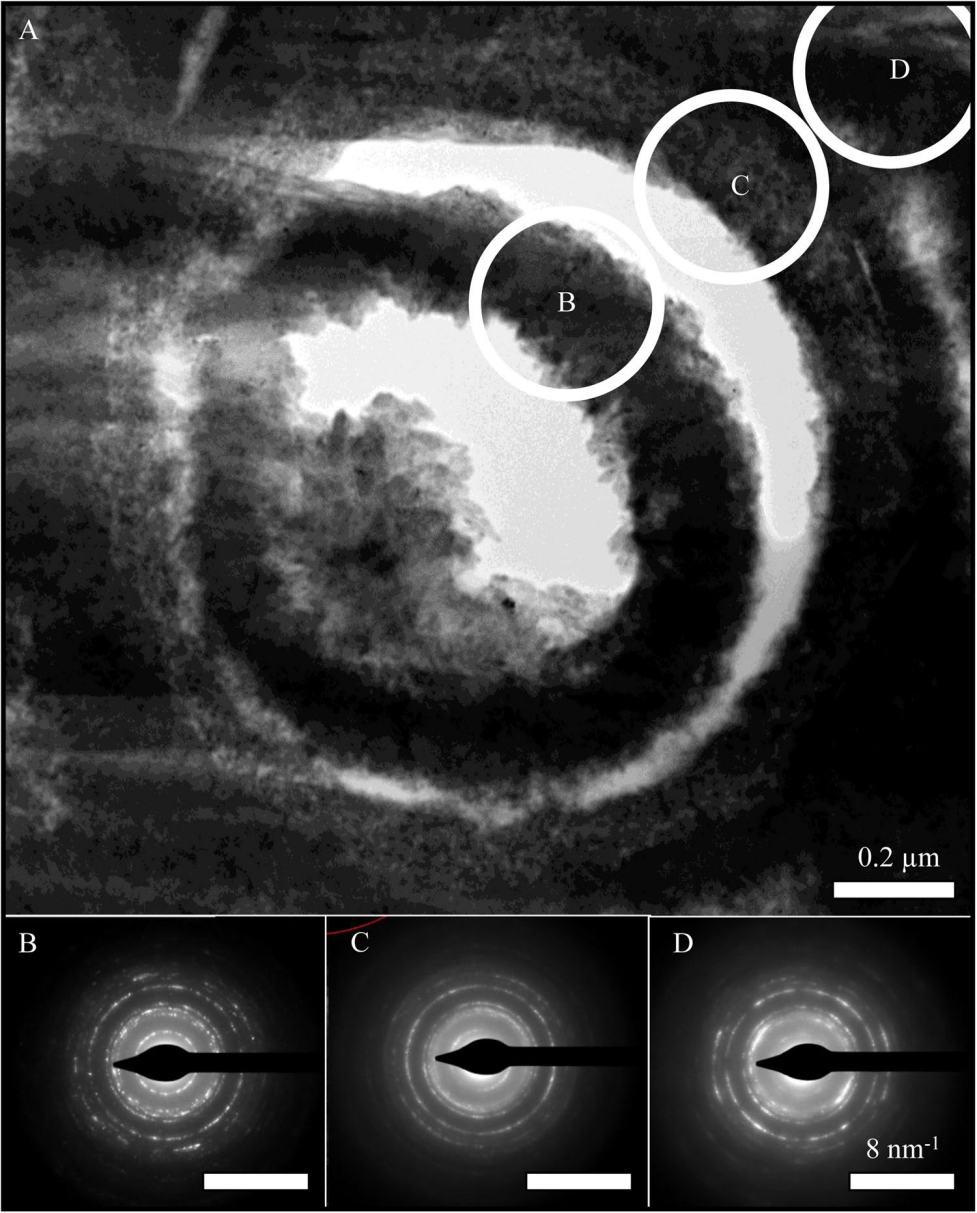
(**A**) Bright field transmission electron micrograph of a TEM lamella containing a mineralised cell envelope. Selected area electron diffraction patterns could not conclusively distinguish intracellular minerals (**B**), biologically-influenced mineralisation associated with the cell envelope structure (**C**) and minerals within the matrix between fossilised cell envelopes (**D**).

Raman spectroscopy revealed both goethite [$\alpha$-FeOOH] and minor lepidocrocite [$\gamma$-FeOOH] precipitating around cell envelopes, with lepidocrocite minerals forming relatively pure phases in the mineralised regions between microfossils (Fig. 5). Transmission electron microscopy indicated that there was no clear mineralogical difference between iron oxide minerals that precipitated in association with the cell envelopes structures and those that precipitated within the matrix (Fig. 6). A transmission-based microXRD experiment conducted at Advanced Light Source (Microdiffraction Beamline 12.3.2) also did not reveal a clear mineralogical biosignature associated with the iron oxide encrusted microfossils (data not shown).

**Figure 7:**
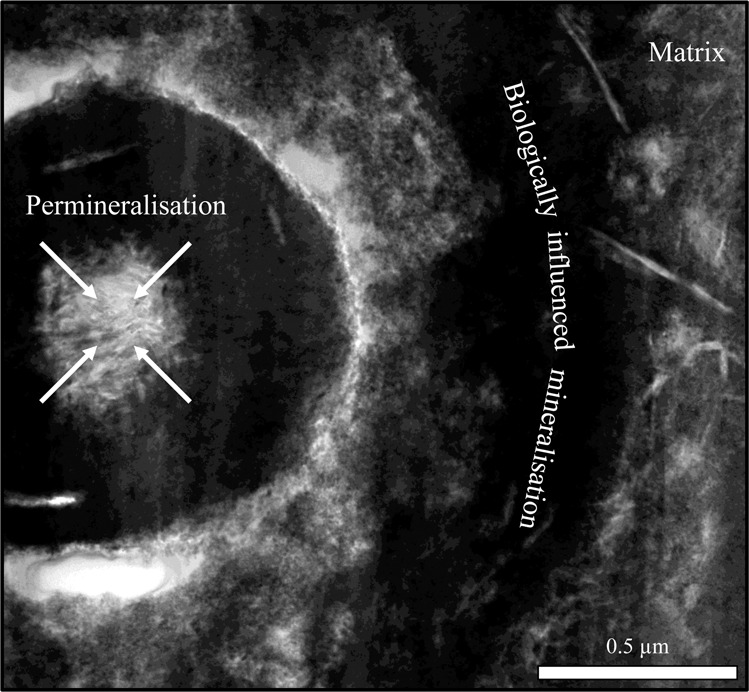
Bright field transmission electron micrograph of fossilised cells highlight that iron oxide minerals follow the direction of the cell envelope structure. In contrast, secondary mineralisation that infills microfossils to form permineralised fossils grow from the edge of the cell envelope structure towards to the centre of the intracellular void (arrows indicate directionality). Video 1. 3D reconstruction of mineral encrusted cell envelopes that have been preserved and infilled pore spaces within a secondary goethite-rich vein that crosscuts the saprolite of banded iron formations at Gandarela, Quadrilátero Ferrífero, Minas Gerais, Brazil.

Transmission electron microscopy of microfossils revealed that biologically influenced mineralisation (BIFM) of the cell envelope appears to follow the direction of the cell envelope structure (Figs. 6–7). In contrast, iron oxide minerals that precipitate in the intracellular regions grow towards the centre of the remnant cell, permineralising the fossil (Fig. 6). These post-fossilisation mineral precipitates appear to be larger than the iron oxide minerals associated with the cell envelope (Fig. 6).

## Discussion

Here, we present abundant mineralised cell envelope structures fossilised within a goethite-rich vein structure that cross-cuts a weathering BIF^6^. These indisputable microbial fossils provide a superb opportunity to understand potential mineralogical biosignatures preserved during microbial fossilisation by authigenic minerals. Rock fissures, fractures and pore spaces present ideal locations for the preservation of microorganisms as elements in solution percolate throughout the weathering profile. The multivalent oxidation states of iron and its low solubility in circumneutral pH environments make iron-rich regions an attractive target for microbial fossilisation in near-surface environments, both on Earth and potentially Mars.

Cell envelope structures reduce the stability of cations in solution, altering the mineral phases that would otherwise have precipitated abiotically^13^. If these biominerals resist recrystallisation, unique mineral phase distributions may be preserved as mineralogical biosignatures^3,14^. In this study, crystal orientation and crystal size of the microbially-influenced iron oxide minerals appears to be altered by the microbial cell envelopes (Figs. 1 and 2) but no clear mineralogical biosignatures were preserved (Figs. 5–7). Although characterisation techniques used here are not exhaustive, there was no clear mineralogical difference between iron oxides that precipitated around the cell envelope and neighbouring iron oxide minerals in the matrix (Figs. 6–7), despite the apparent enrichment of aluminium associated with the cell envelope (Fig. 3). Therefore, the organic-mineral complexes that resist recrystallisation during the initial stages of mineral formation^15^ do not appear to be preserved in million-year-old fossils. Given the low-temperature formation and alteration of the iron oxide minerals^9^, the aliphatic hydrocarbon moieties preserved with the microfossils are likely to be replaced during low-temperature metasomatism by iron and aluminium in solution. Levett *et al.*^6^ demonstrated that organic biosignatures associated with younger (ca. 2 Ma) permineralised microfossils in the overlying duricrust are completely replaced, likely due to the increased cycling of iron oxide minerals that occurs near the surface^16^.

In this study, mineral precipitates associated with cell envelope structures are extremely fine-grained compared with the post-death intracellular mineral precipitates (Figs. 6–7). Cations binding to active sites on the cell envelope^17^ appears to create multiple mineral nucleation sites, restricting crystal growth. In contrast, post-death mineral precipitates within the intracellular voids have fewer mineral nucleation sites and, therefore, are allowed to grow in a less restricted manner. Consistent with Cosmidis, *et al*.^18^, intracellular mineral precipitates always grow from the cell envelope inward to fill the intracellular void.

The 3D characterisation of iron oxide encrusted microbial fossils, provides an important opportunity to produce orientated reconstructions useful in the search for fossilised bacteria or biofilm (Video 1). Though FIB-SEM was used in this study, synchrotron-based nanotomography now offers non-destructive 3D reconstructions with submicron spatial resolution^19^. These technical developments offer unparalleled opportunities to understand the mechanisms that contribute to microbial fossilisation. The 100 nm resolution of the 3D reconstruction produced for multiple sections of each microbial fossil. The microfossils examined here are typically cocci-shaped and approximate 1 μm in diameter. *Sarcina*-like multicellular packet structures (for example, Supplementary Fig. S3) are never observed in the fossilised biofilm characterised here; however, rarely, paired cells are preserved that share a cytoplasm in a single section (Fig. 2). These textures provide evidence that the fossilised microorganisms presented here may be able to replicate during the initial stages of biomineralisation as has been previously postulated ^20–22^.

Microorganisms fossilised by authigenic mineral nucleation, rather than the binding of sediments within the biofilm^23^, provide valuable insights into the environmental conditions in which the living microorganism existed. These biogenic minerals may also provide information on influence of the microorganisms on their surrounding environment. Previous experiments have demonstrated the precipitation of lepidocrocite associated with neutrophilic iron-oxidising microorganisms^24^ and nitrate-dependent iron-oxidising bacteria^25^. Given the microfossil structures in this study are generally cocci-shaped, they are unlikely to represent sheath structures of classic *Leptothrix*-type neutrophilic iron-oxidising bacteria^26^, though filaments do exist (see Video 1; 38–40 s, top right-hand corner). In addition, the low nitrogen availability in these environments^27^ suggests that microfossils are also unlikely to represent nitrate-dependant iron oxidisers. The apparent binding of aluminium with cell envelope structures, indicates that these microorganisms were likely to have been preserved by the passive nucleation of minerals on the cells’ surfaces^14,28^. The formation of lepidocrocite together with goethite (Fig. 5) suggests that the pH was between 5–7. Lepidocrocite forms preferentially to goethite under slightly slower oxidation rates of iron^29^, indicating reduced partial pressures of oxygen in pore spaces below the surface compared with atmospheric conditions.

In this BIF weathering profile, many cells contribute to mineral nucleation by the passive interaction of cations with the net negative cell envelope^28^. Even amongst the cells that contribute to biomineralisation^30^, few are likely to achieve a state of ‘fossilisation’, whereby they are preserved in the geological record. Extensive mineralisation is required to achieve microbial fossilisation and preservation. Based on earlier findings^6^, aluminium binding irreversibly with cell envelope structures appears to play an important role in the preservation of organic biosignatures; however, synchrotron-based FT-IR analysis could not resolve aluminium-organic complexes.

The preservation of microorganisms in the geological record is rare. As such, an abundance of well-preserved microfossils in any environment requires careful consideration. To resist the breakdown of cellular components, particularly cell envelope structures and potentially EPS, rapid and extensive mineralisation is required. The influence of water in microbial fossilisation within the lithosphere is also likely to be critical. While fine-grained, generally amorphous iron oxide precipitates readily nucleate on cell envelope structures in iron-rich aqueous environments^28^, for example, 2-line ferrihydrite^31^; these cells are unlikely to be fossilised within water saturated environments. Therefore, following this initial stage of biomineralisation during a cells exposure to cation-rich solutions, periods of drying appear to be imperative for fossilisation. During dehydration, any remaining ions in solution (in this case, predominately iron and aluminium), would be concentrated, accelerating additional mineral nucleation on the cells’ surfaces. Alternating wet and dry periods may be required to promote additional mineralisation. In this scenario, additional metals in solution would be provided during wet periods, which may allow for the recrystallisation of existing iron oxide minerals and the additional precipitation of new iron oxide minerals^32^. During drying periods, newly mineralised microorganisms may be committed to the geological record, contributing to preservation of relatively large microfossil clusters.

The organic compounds associated with the cell envelopes are likely to be preserved by the electrostatic-driven nucleation of aluminium and iron oxide minerals within relatively oxidising environments^6,7,28^. As the microfossils are continuously exposed to aluminium and iron-rich solutions, the mineralised cell envelopes appears to act as a filter; iron is allowed into the cell whereas aluminium is enriched around the cell envelope^6,33^. The structure of the cell envelop appears to restrict aluminium transfer into the cell^33^, possibly even after cell death. Aluminium may also continue to be enriched around the cell envelope as it preferentially precipitates with existing aluminium-substituted iron oxide minerals that have previously nucleated on the cells’ surfaces^29^. In this way, even after all the organic components of the cell envelope are replaced and the cell has been completely permineralised, aluminium enrichment around the cells may help to preserve the bacteriomorphic structure within the geologic record^6,34^.

Appropriate sample preparation for high-resolution analytical work is critical. Many techniques require a polished surface to spatially resolve distinctions between minerals influenced by microorganisms compared with ‘abiotic’ mineral precipitates. To study microfossils, sample preparation using a FIB-SEM offers a number of benefits including, targeted preparation of localised regions of interest without introducing organic contaminants. As a destructive sample preparation technique, great care and skill is required when preparing microfossil lamella using a FIB-SEM; however, this sample preparation technique is highly versatile^10^. Samples can be made thin enough to be analysed using transmission X-ray and infrared sources but also robust enough for NanoSIMS, a destructive secondary ion technique. Therefore, FIB-SEM sample preparation allows for highly targeted, polished sample preparation and for correlation between several different analytical datasets, as demonstrated in this study. Ultrathin samples (~100 nm thick) can also be prepared for high-resolution transmission electron microscopy (HR-TEM) and scanning transmission X-ray microscopy.

The redistribution of elements that contribute to near-surface microbial fossilisation in rock pore spaces and fissures is fundamental to targeting drill regions for the identification of microfossils on samples from Mars. Organic biosignatures and abundant microbial fossils preserved in iron-rich environments highlights the potential to target iron-rich regions on the Martian surface for the search of potential microbial biosignatures. In depth characterisation of indisputable microbial fossils combining a suite of nano- and microscale analytical techniques sets important benchmarks for the identification of biosignatures within the geological record^35^. Additional studies of natural microbial fossils in a variety of environments is required to understand potential biosignatures preserved in different environments and aim to develop new robust biosignatures, for example, mineralogical biosignatures.

## Conclusions

Iron oxide encrusted microfossils, preserved in near-surface environments by the extensive mineralisation of the cell envelope structures, prevent the degradation of the soft organic material of microorganisms. A remarkable variety of microfossil textures are preserved. Following cell envelope mineralisation, continued exposure to iron-rich solutions results in the permineralisation of microfossils, as iron oxide minerals grow inwards to infill intracellular voids. Microorganisms appear to be able to replicate even while partially mineralised. As microorganisms are fossilised, they can infill pore and fissure spaces within near-surface rocks. These iron oxide plateaus that contain abundant microbial fossils on Earth may also be attractive geomorphological targets for the search for life, or remnants of life (fossils) on Mars.

## Supplementary information

Supplementary Information.

## Data Availability

All data Raman data is available at https://drive.google.com/open?id=1Q46V1L0bXVJG9h9iBJVCzk_lAirKdEUG.

## References

[1] McKay DS (1996). Search for past life on Mars: possible relic biogenic activity in Martian meteorite ALH84001. Science.

[2] Davila, A. F., Fairén, A. G., Schulze-Makuch, D. & McKay, C. P. In *From Fossils to Astrobiology: Records of Life on Earth and Search for Extraterrestrial Biosignatures* (eds Joseph Seckbach & Maud Walsh) 471-489 (Springer Netherlands, 2008).

[3] Banfield JF, Moreau JW, Chan CS, Welch SA, Little B (2001). Mineralogical biosignatures and the search for life on Mars. Astrobiology.

[4] McMahon S (2018). A field guide to finding fossils on Mars. J. Geophys. Res. Planets.

[5] Westall F (1999). The nature of fossil bacteria: a guide to the search for extraterrestrial life. J. Geophys. Res. Planets.

[6] Levett A (2019). The role of aluminium in the preservation of microbial biosignatures. Geosci. Front..

[7] Monteiro H, Vasconcelos P, Farley K (2018). A Combined (U‐Th)/He and Cosmogenic 3He Record of Landscape Armoring by Biogeochemical Iron Cycling. J. Geophys. Res. Earth.

[8] Monteiro H, Vasconcelos P, Farley K (2018). A combined (U‐Th)/He and cosmogenic ^3^He record of landscape armoring by biogeochemical iron cycling. J. Geophys. Res. Earth.

[9] Monteiro, H. D. S. Paleoenvironmental evolution of continental landscapes through combined high-resolution geochronology and δ 18O ion microprobe analysis of goethite. (2017).

[10] Heaney PJ, Vicenzi EP, Giannuzzi LA, Livi KJ (2001). Focused ion beam milling: A method of site-specific sample extraction for microanalysis of Earth and planetary materials. Am. Mineral..

[11] Hanesch M (2009). Raman spectroscopy of iron oxides and (oxy) hydroxides at low laser power and possible applications in environmental magnetic studies. Geophys. J. Int..

[12] Brandt N, Brovko O, Chikishev AY, Paraschuk O (2006). Optimization of the rolling-circle filter for Raman background subtraction. Appl. Spectrosc..

[13] Levett A (2020). Biogeochemical cycling of iron: implications for biocementation and slope stabilisation. Sci. Total Environ..

[14] Li J, Benzerara K, Bernard S, Beyssac O (2013). The link between biomineralization and fossilization of bacteria: insights from field and experimental studies. Chem. Geol..

[15] Banfield JF, Welch SA, Zhang H, Ebert TT, Penn RL (2000). Aggregation-based crystal growth and microstructure development in natural iron oxyhydroxide biomineralization products. Science.

[16] Monteiro HS, Vasconcelos PM, Farley KA, Spier CA, Mello CL (2014). (U–Th)/He geochronology of goethite and the origin and evolution of cangas. Geochim. Cosmochim. Ac..

[17] Fein JB, Daughney CJ, Yee N, Davis TA (1997). A chemical equilibrium model for metal adsorption onto bacterial surfaces. Geochim. Cosmochim. Ac..

[18] Cosmidis J (2013). Nanometer-scale characterization of exceptionally preserved bacterial fossils in Paleocene phosphorites from Ouled Abdoun (Morocco). Geobiology.

[19] Müller S (2018). Quantification and modeling of mechanical degradation in lithium-ion batteries based on nanoscale imaging. Nat. Commun..

[20] Phoenix V, Konhauser K (2008). Benefits of bacterial biomineralization. Geobiology.

[21] Phoenix VR, Adams DG, Konhauser KO (2000). Cyanobacterial viability during hydrothermal biomineralisation. Chem. Geol..

[22] Benzerara K (2011). Significance, mechanisms and environmental implications of microbial biomineralization. C. R. Geosci..

[23] Newman SA, Mariotti G, Pruss S, Bosak T (2016). Insights into cyanobacterial fossilization in Ediacaran siliciclastic environments. Geology.

[24] Chan CS, Fakra SC, Emerson D, Fleming EJ, Edwards KJ (2011). Lithotrophic iron-oxidizing bacteria produce organic stalks to control mineral growth: implications for biosignature formation. ISME J..

[25] Larese-Casanova P, Haderlein SB, Kappler A (2010). Biomineralization of lepidocrocite and goethite by nitrate-reducing Fe(II)-oxidizing bacteria: Effect of pH, bicarbonate, phosphate, and humic acids. Geochim. Cosmochim. Ac..

[26] Emerson D, Ghiorse WC (1992). Isolation, cultural maintenance, and taxonomy of a sheath-forming strain of *Leptothrix discophora* and characterization of manganese-oxidizing activity associated with the sheath. Appl. Environ. Microbiol..

[27] Messias M, Leite M, Meira Neto J, Kozovits A, Tavares R (2013). Soil-vegetation relationship in quartzitic and ferruginous Brazilian rocky outcrops. Folia Geobot..

[28] Ferris FG, Fyfe WS, Beveridge TJ (1988). Metallic ion binding by *Bacillus subtilis*: implications for the fossilization of microorganisms. Geology.

[29] Cornell, R. M. & Schwertmann, U. The iron oxides: structure, properties, reactions, occurrences and uses. (John Wiley & Sons, 2003).

[30] Levett A, Gagen EJ, Southam G (2019). Small but mighty: microorganisms offer inspiration for mine remediation and waste stabilisation. Microbiol. Aust..

[31] Kennedy C, Scott S, Ferris F (2004). Hydrothermal phase stabilization of 2-line ferrihydrite by bacteria. Chem. Geol..

[32] Legrand L, Mazerolles L, Chaussé A (2004). The oxidation of carbonate green rust into ferric phases: solid-state reaction or transformation via solution. Geochim. Cosmochim. Ac..

[33] Londono SC, Hartnett HE, Williams LB (2017). Antibacterial activity of aluminum in clay from the Colombian Amazon. Environ. Sci. Technol..

[34] Levett A (2016). Evidence of biogeochemical processes in iron duricrust formation. J. S. Am. Earth Sc..

[35] Levett A (2020). Microbial weathering signatures in lateritic ferruginous duricrusts. Earth Planet. Sci. Lett..

