# Supplementary material for: Characterisation of iron oxide encrusted microbial fossils
Source: Sci Rep. 2020 Jun 18;10:9889. doi: 10.1038/s41598-020-66830-z (PMC7303173; doi:10.1038/s41598-020-66830-z)
Supplement: Supplementary file 1 — Supplementary Information. [file 41598_2020_66830_MOESM1_ESM.pptx]

## Slide 1
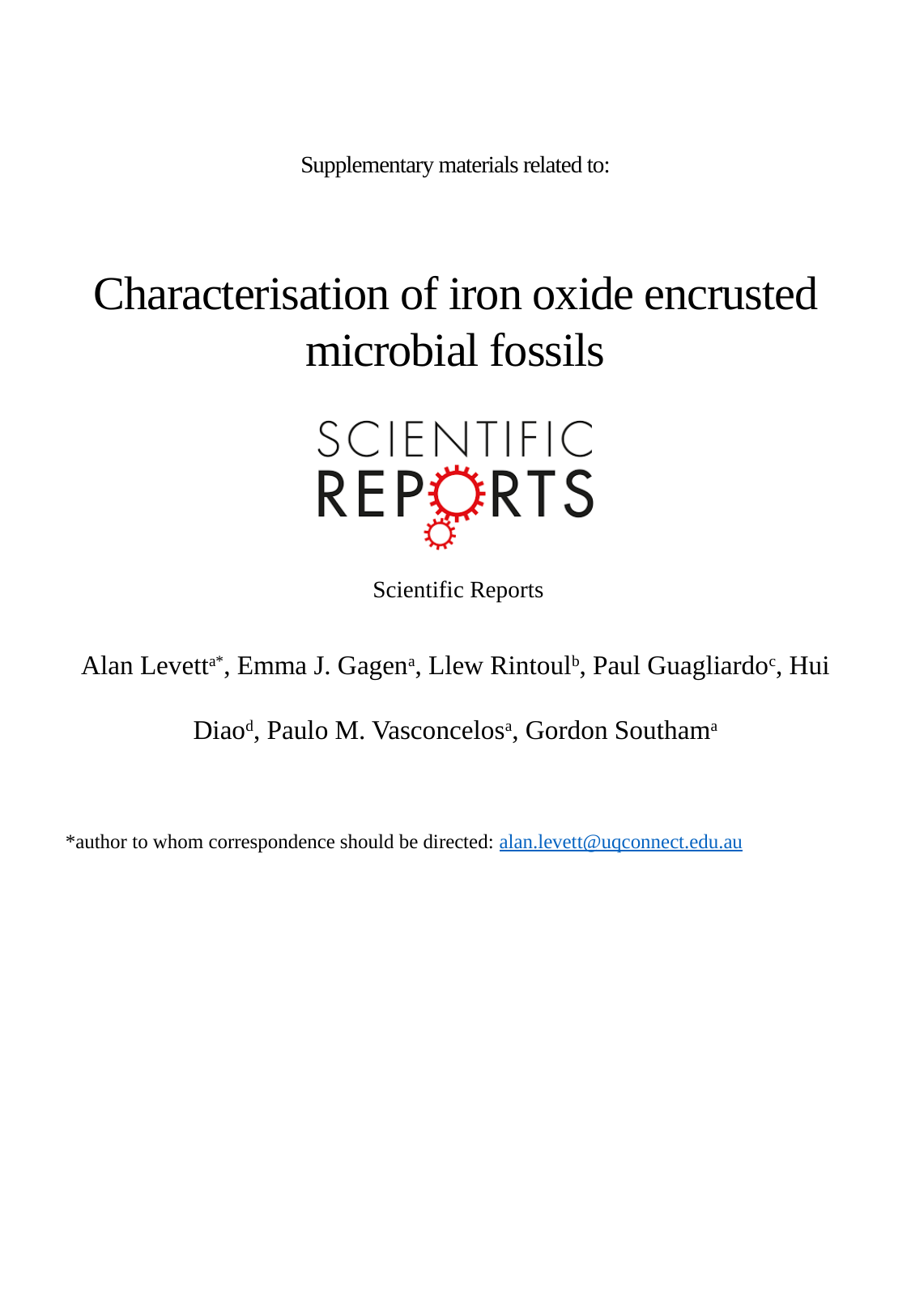

Supplementary materials related to:
Characterisation of iron oxide encrusted microbial fossils
 Scientific Reports
Alan Levetta*, Emma J. Gagena, Llew Rintoulb, Paul Guagliardoc, Hui Diaod, Paulo M. Vasconcelosa, Gordon Southama
*author to whom correspondence should be directed: alan.levett@uqconnect.edu.au

## Slide 2
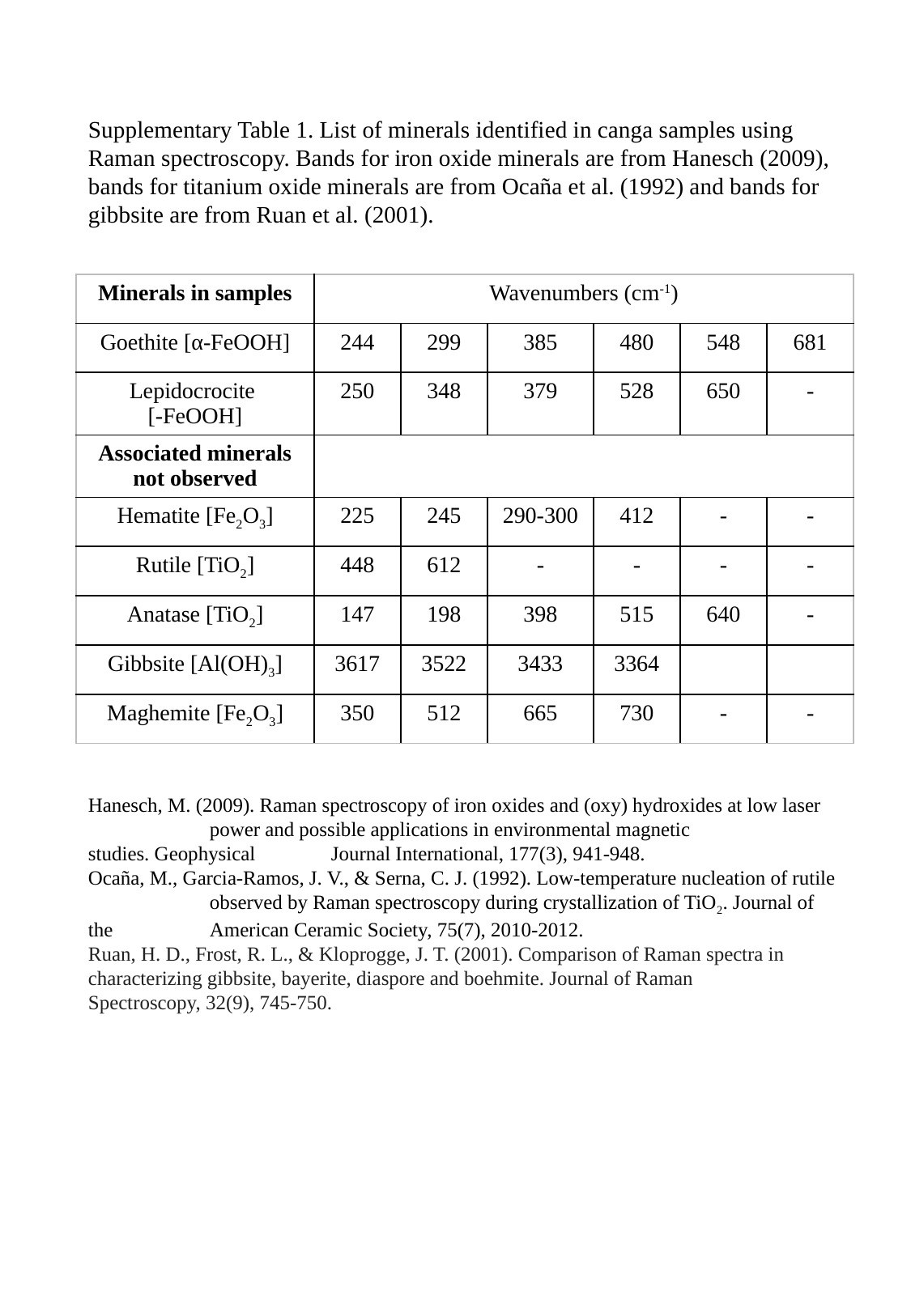

Supplementary Table 1. List of minerals identified in canga samples using Raman spectroscopy. Bands for iron oxide minerals are from Hanesch (2009), bands for titanium oxide minerals are from Ocaña et al. (1992) and bands for gibbsite are from Ruan et al. (2001).
Hanesch, M. (2009). Raman spectroscopy of iron oxides and (oxy) hydroxides at low laser 	power and possible applications in environmental magnetic studies. Geophysical 	Journal International, 177(3), 941-948.
Ocaña, M., Garcia‐Ramos, J. V., & Serna, C. J. (1992). Low‐temperature nucleation of rutile 	observed by Raman spectroscopy during crystallization of TiO2. Journal of the 	American Ceramic Society, 75(7), 2010-2012.
Ruan, H. D., Frost, R. L., & Kloprogge, J. T. (2001). Comparison of Raman spectra in 	characterizing gibbsite, bayerite, diaspore and boehmite. Journal of Raman 	Spectroscopy, 32(9), 745-750.

## Slide 3
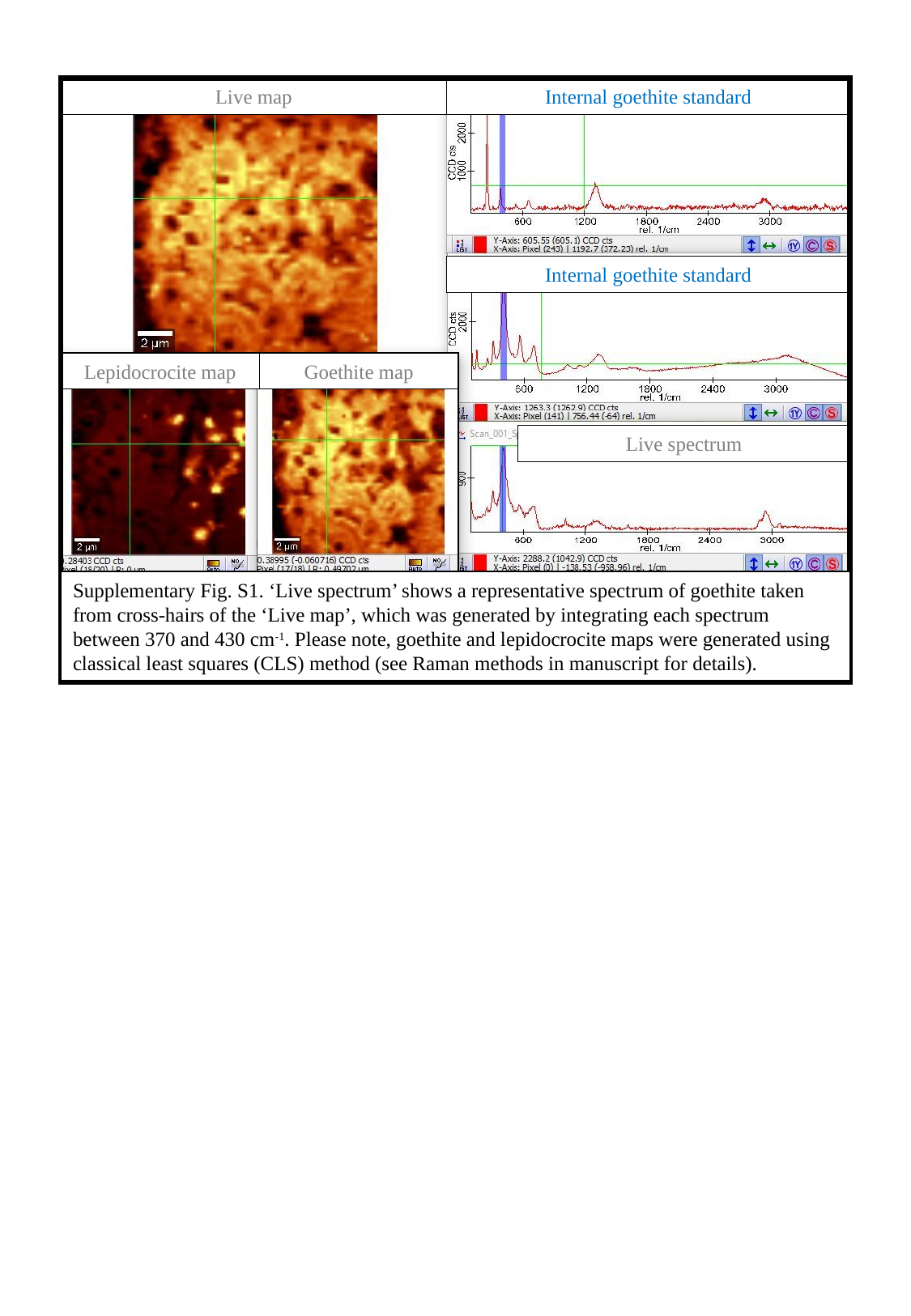

Live map
Internal goethite standard
Internal goethite standard
Lepidocrocite map
Goethite map
Live spectrum
Supplementary Fig. S1. ‘Live spectrum’ shows a representative spectrum of goethite taken from cross-hairs of the ‘Live map’, which was generated by integrating each spectrum between 370 and 430 cm-1. Please note, goethite and lepidocrocite maps were generated using classical least squares (CLS) method (see Raman methods in manuscript for details).

## Slide 4
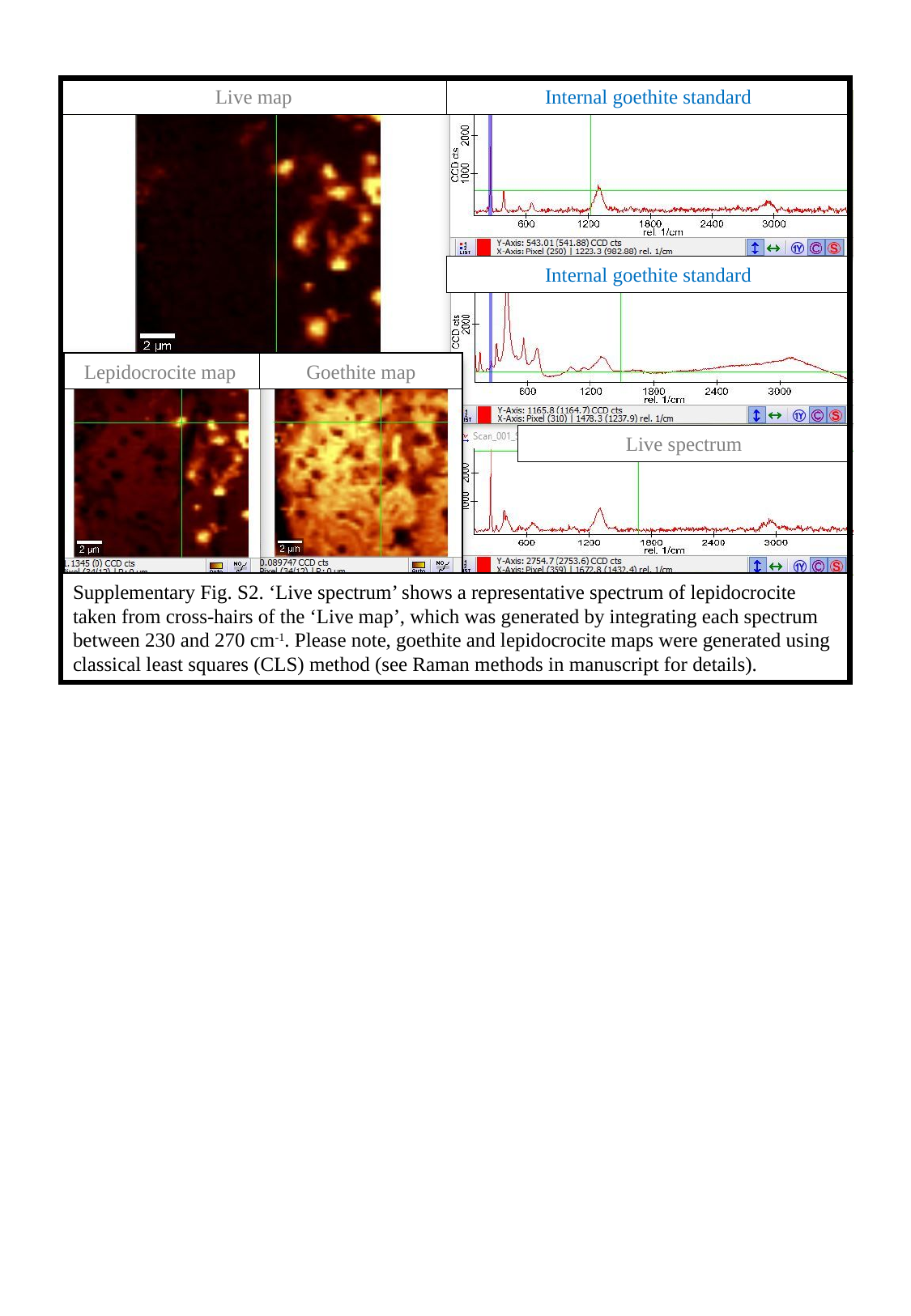

Live map
Internal goethite standard
Internal goethite standard
Lepidocrocite map
Goethite map
Live spectrum
Supplementary Fig. S2. ‘Live spectrum’ shows a representative spectrum of lepidocrocite taken from cross-hairs of the ‘Live map’, which was generated by integrating each spectrum between 230 and 270 cm-1. Please note, goethite and lepidocrocite maps were generated using classical least squares (CLS) method (see Raman methods in manuscript for details).

## Slide 5
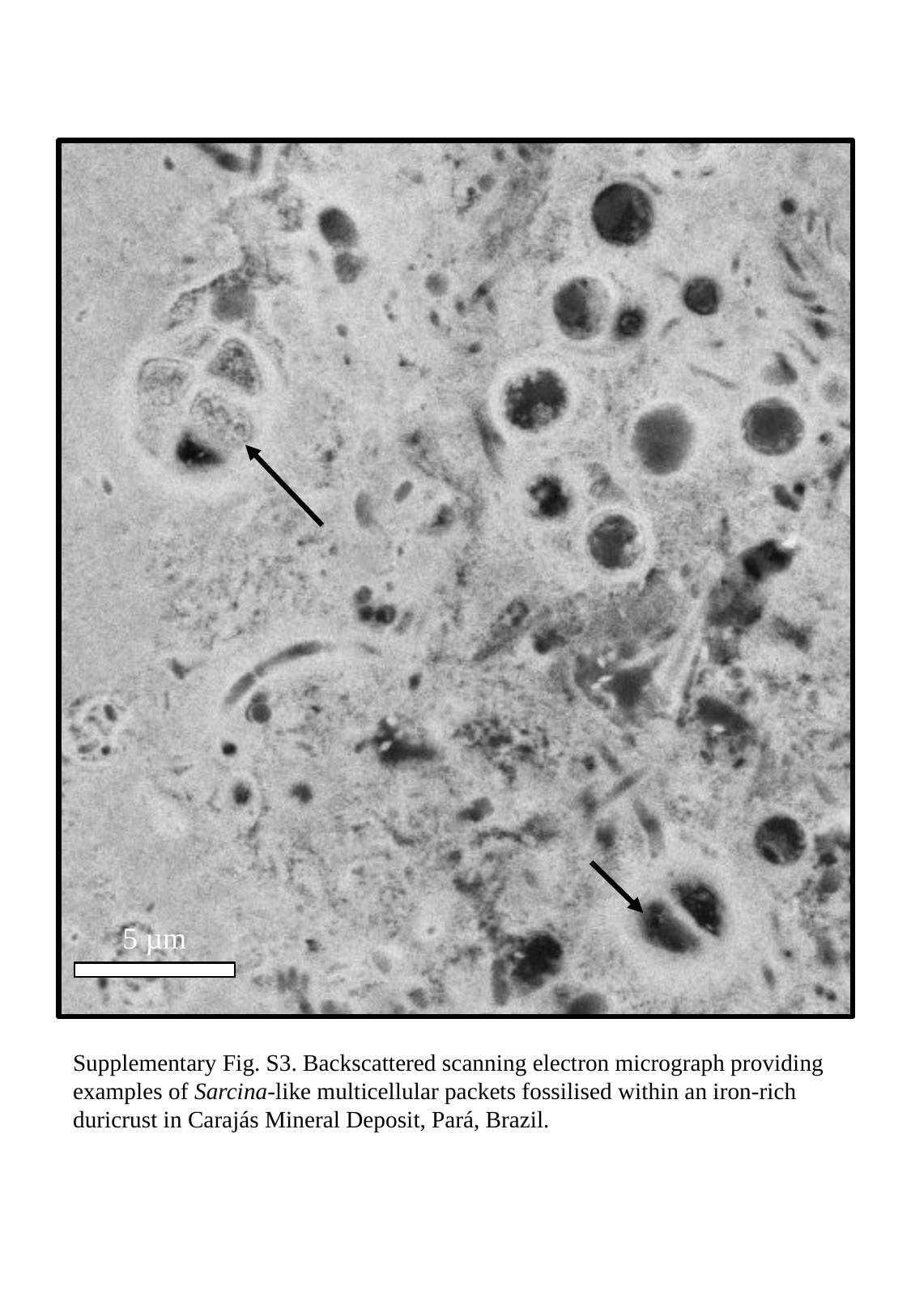

5 µm
Supplementary Fig. S3. Backscattered scanning electron micrograph providing examples of Sarcina-like multicellular packets fossilised within an iron-rich duricrust in Carajás Mineral Deposit, Pará, Brazil.
